# The Inspection of Public Institutions

**Published:** 1914-05-30

**Authors:** 


### The Hospital

**The Workers' Newspaper of Administrative Medicine and Institutional Life, Administration, National Insurance and Health.**

No. 1457, Vol. LVI. SATURDAY, MAY 30, 1914. PRICE ONE PENNY.

### THE INSPECTION OF PUBLIC INSTITUTIONS.

We have often been struck by the incompleteness of inspectors' reports of public institutions, and by their failure to call attention to defects which are found glaringly present, together with many matters which should not fail to find a place in such reports, if the inspectors invariably were fully competent, energetic, fearless, and independent. We have before us at the present time the case of the North Evington Infirmary at Leicester, a report on which by Sir Henry Burdett appears on another page of our present issue. A remarkable fact in this connection is brought out in the report in the *Leicester Post* of the 20th instant of a meeting of the Guardians, when Mr. Sherriff asked the chairman what group of Guardians held that the maximum salary for a medical superintendent should be £500 anywhere? The chairman replied he had not the slightest idea where the statement came from. In fact we happen to know that the chairman within the last three months has discussed this point for fully a quarter of an hour at a private conference, and dwelt upon the insistence of a section of the Guardians that the salary should be £450, as evidence of the great difficulty there was in securing a large enough salary to attract a really competent medical superintendent. Similarly, the visiting medical officer, Dr. John Dodd, in a long letter to the *Leicester Post* published on the 23rd instant, whilst inferentially supporting the present condition of affairs at North Evington and condemning Sir Henry's report on the ground that he did not seek—and we presume adopt?—the official view, does in fact uphold the material points in his report, though he exhibits a want of accurate knowledge himself in regard to the actual condition of the maternity department, and of certain nursing matters which Sir Henry satisfied himself existed, as the position of affairs he found on his visit demonstrated.

The fact is, we take it, that throughout the Poor-Law service, until the creation of the modern up-to-date Poor-Law infirmary, the motto inculcated into officials and upheld by many Boards of Guardians was that of "good enough" when applied to any existing evil or anomaly, because the poor inmates are paupers and belong to the very poorest members of the population. This baneful atmosphere is so demoralising that many experienced administrators throughout our hospital system find it necessary not to take into their service porters and other officials who have served under the Poor-Law. How far this remnant of the worst days of Poor-Law relief clings still to some of the higher officials, and whether or not it permeates directly or indirectly, and possibly unconsciously, the minds of even Poor-Law inspectors, are points well worthy of their consideration. At any rate, it is a curious fact that in a town like Leicester, where the voluntary medical institutions are remarkable for their efficiency and thoroughness, both in regard to administration and the treatment and care of patients, the chairman of the Committee of Guardians responsible for the Poor-Law infirmary, in dealing with Sir Henry Burdett's report, claimed that "if the Local Government Board had not asked them to stay their hand every suggestion made in that report would have been carried out long ago. It is the Local Government Board and not the Board of Guardians which must be blamed for the present deficiencies at North Evington." We hope the committee to which Sir Henry Burdett's report has been referred will examine fully this remarkable statement, and that the Local Government Board may be moved to explain matters from their point of view.

We have gone fully into the case of North Evington because it illustrates the great difficulties in which inspection may land the inspector. Many hospital committees up and down the country, and many Boards of Guardians, find themselves handicapped by the fact that they have no member or members who really understand the technical side of hospital administration and hospital work. Consequently, they consider when an inspection is made that the inspector should be accompanied by representatives of the governing body and the official staff, and they are apt to feel hurt when the honour of so flattering a reception is declined.

for the purely technical reason that the inspection of a hospital or Poor-Law infirmary is a matter of grave importance involving a thorough examination of the relative efficiency or otherwise of each department, and the causes of any deficiency which may be found to exist. The inspector, therefore, who has the necessary knowledge and experience to undertake so responsible and arduous a task is bound in honour to decline, until after his inspection is completed, the attendance of a number of officials and committee-men. The more efficient and knowledgeable the individual members of the staff of a public institution are, and the keener their efforts to make their institution stand out as up to date and admirably administered, the more gladly do they welcome and appreciate the visit of an inspector of repute.

When Sir Henry Burdett started on his inspections of the voluntary hospitals and Poor-Law infirmaries of England and Wales, now nearly completed, he was urged by the press to do the work thoroughly and to be fearlessly outspoken in his reports. This policy has been pursued, and it has won the cordial co-operation of the authorities and the staffs, whilst it has produced effects which cannot fail to leave their mark upon the voluntary hospital system for many years to come. The whole object of inspections which are to be thoroughly effective and valuable must be to make it possible for the community which each institution serves to realise its actual condition and relative efficiency, and at the same time to learn what is wanting, if anything be wanting, to improve one or both of these important essentials. So the inspector, when he does the more serious portions of his work, must pay his visits unannounced and take the institution and test its work as they in fact are. Any ceremonial or official observances which a committee or Board of Guardians may desire, such as an inspection of the wards or parts of the buildings in their company, can only properly be made after the technical inspection has been first completed.

